# Acute effects of taxanes on sub-tissue fluid proportions in patients with breast cancer who underwent taxane-based chemotherapy

**DOI:** 10.1007/s00520-026-10612-2

**Published:** 2026-03-29

**Authors:** Alper Tuğral, Yeşim Bakar, Murat Akyol

**Affiliations:** 1 Faculty of Health Sciences, Department of Physiotherapy and Rehabilitation, Bakircay University, Izmir, Turkey; 2 Faculty of Medicine, Department of Medical Oncology, Bakircay University, Izmir, Turkey

**Keywords:** Breast cancer, Breast cancer-related lymphedema, Tissue dielectric constant, Chemotherapy

## Abstract

**Purpose:**

Breast cancer-related lymphedema (BCRL) is one of the most burdensome chronic side effects of breast cancer treatment. This study aimed to analyze the acute effects of taxane-based CT on the potential BCRL among unilateral breast cancer (BC) patients by using the tissue dielectric constant (TDC) method.

**Methods:**

The TDC method was performed via MoistureMeterD (DelfinTech, Finland) which generates a 300-MHz electromagnetic wave in the control unit and is transmitted via a probe. Both upper extremities were assessed in the predefined reference points (arm, forearm, thorax, and web space) via different penetration depths from 0.5 to 5.0 mm probes before and after the completion of chemotherapy.

**Results:**

A total of 51 BC patients were included in this study. Twelve and 39 patients were referred to neoadjuvant chemotherapy (NACT) and adjuvant chemotherapy (ACT), respectively. In the adjuvant CT, the web space reference point was found to have significantly higher TDC value in 0.5-, 1.5-, and 2.5-mm probe depths after CT. In the NACT, no significant change was found before and after CT in all reference points except for the affected site thorax reference point.

**Conclusion:**

This study showed that there was no significant acute increase in inter-arm TDC ratios after taxane-based chemotherapy. However, the forearm and web space should be thoroughly monitored in patients to discriminate against BCRL who underwent adjuvant CT since the potential BCRL and peripheral edema due to taxanes might have affected each other.

## Introduction

Breast cancer (BC) is the most common type of cancer among women globally. According to the GLOBOCAN report, the incidence of BC reaches nearly 12% [1]. However, recent advances have improved disease-free survival rates by up to 90% for 5 years among BC patients [2].

The treatment of BC is comprised of different modalities such as surgery, chemotherapy, radiotherapy, and hormone therapy. Thus, the experienceable side effects of BC treatment vary remarkably among BC survivors [3]. Fatigue, musculoskeletal complaints, and diminished functional capacity can commonly be seen in BC patients not only during the primary treatment but also in the long-term continuum [4]. Breast cancer-related lymphedema (BCRL), which is characterized by the chronic accumulation of protein-rich fluid in interstitial spaces due to surgical excision of axillary lymph nodes and other contributing factors, is a debilitating chronic condition among BC survivors [5]. Since BCRL cannot be treated completely, early diagnosis of BCRL can prevent aggravation of excessive swelling. Though swelling is the main symptom of BCRL, increased heaviness feeling may trigger deteriorated upper extremity function; advanced skin changes such as fibrosis and musculoskeletal pain can also worsen the clinical table [6]. Therefore, early diagnosis of changes associated with the BCRL is of utmost importance to preserve and reach maximal clinical outcomes [7].

To date, many risk factors have been reported for BCRL such as axillary lymph node dissection (ALND), regional radiotherapy, higher body mass index (BMI), age, and chemotherapy [8]. Among these, taxane-based chemotherapy, despite its proven efficacy and widespread use, has raised concerns about its potential role in the development of BCRL. While taxane-based chemotherapy has been reported to be unrelated to the development of BCRL except for mild swelling in peripheral tissues [9], other studies have suggested that taxane-based chemotherapy, particularly adjuvant docetaxel, may significantly increase the risk of BCRL [10, 11]. Although many researchers used circumference measurement and symptomatologic assessment to diagnose early BCRL in line with the literature, those are also known as having the potential inability to catch small differences in the tissue in the early phases of BCRL. In addition, there is no gold standard to diagnose BCRL; the varying rates of risk of BCRL can also be attributed to this situation [12].

The tissue dielectric constant (TDC) method has been well acquainted with the lymphology literature for over the last decade. Since this method can be easily used and repeated locally wherever possible and desired, using its potential diagnostic feature can be advantageous among BC survivors who are at risk of BCRL [7, 13–16]. There are many studies not only that investigate BCRL but also lower limb lymphedema [17–19]. The TDC method was also used in varied conditions from diabetes [20] to decubitus [21].

Since circumference measurement and symptom-based assessment may fail to detect early tissue fluid changes due to the wide range of symptoms experienced by patients with BC during systemic chemotherapy, there is an emerging need for diagnosing potential preclinical BCRL before and after taxane-based chemotherapy. In addition, to the best of our knowledge, the TDC method has not been used to assess the potential effects of taxane-based chemotherapy on tissue fluid accumulation in BC patients. Therefore, we aimed to analyze the acute effects of taxanes by analyzing dielectric values of tissues in the context of potential preclinical BCRL.

## Materials and methods

### Study design

This prospective observational study was held between January and June 2023 with the approval of Izmir Bakircay University Ethical Board of Clinical Studies (806/826/28122022) and followed the Strengthening the Reporting of Observational Studies in Epidemiology (STROBE) guideline [22]. This study was performed according to the 1964 Helsinki Declaration and its later amendments or comparable ethical standards.

### Patients

Patients who were diagnosed with unilateral breast cancer and were scheduled to receive taxane-based neoadjuvant or adjuvant chemotherapy in the medical oncology unit were screened and invited to participate in the study. Being a volunteer to participate, having been diagnosed with malignant BC of the unilateral breast, female gender, and having communication skills in the native language were set as inclusion criteria. Having breast surgery for cosmetic reasons, cognitive and mental deficits, having an active infection, open wound(s), or allergic skin reactions in measurement sites and who were not candidates for systemic chemotherapy were excluded. Since this study was designed as a prospective observational study, all eligible patients who were scheduled to receive taxane-based chemotherapy during the recruitment period were invited to participate. Therefore, the sample size reflects a convenience sample of patients meeting the inclusion criteria during the study period. Patients were categorized as receiving neoadjuvant chemotherapy (NACT) or adjuvant chemotherapy (ACT) according to the treatment setting at the time of the baseline TDC assessment.

### Assessment

#### Demographic data form

A basic information form was applied in which patients’ clinical (type of breast and axillary operation if any, type of chemotherapy regimen, other comorbidities if any) and sociodemographic (age, BMI, dominant site, etc.) characteristics are questioned.

#### Tissue dielectric constant (TDC)

**Fig. 1:**
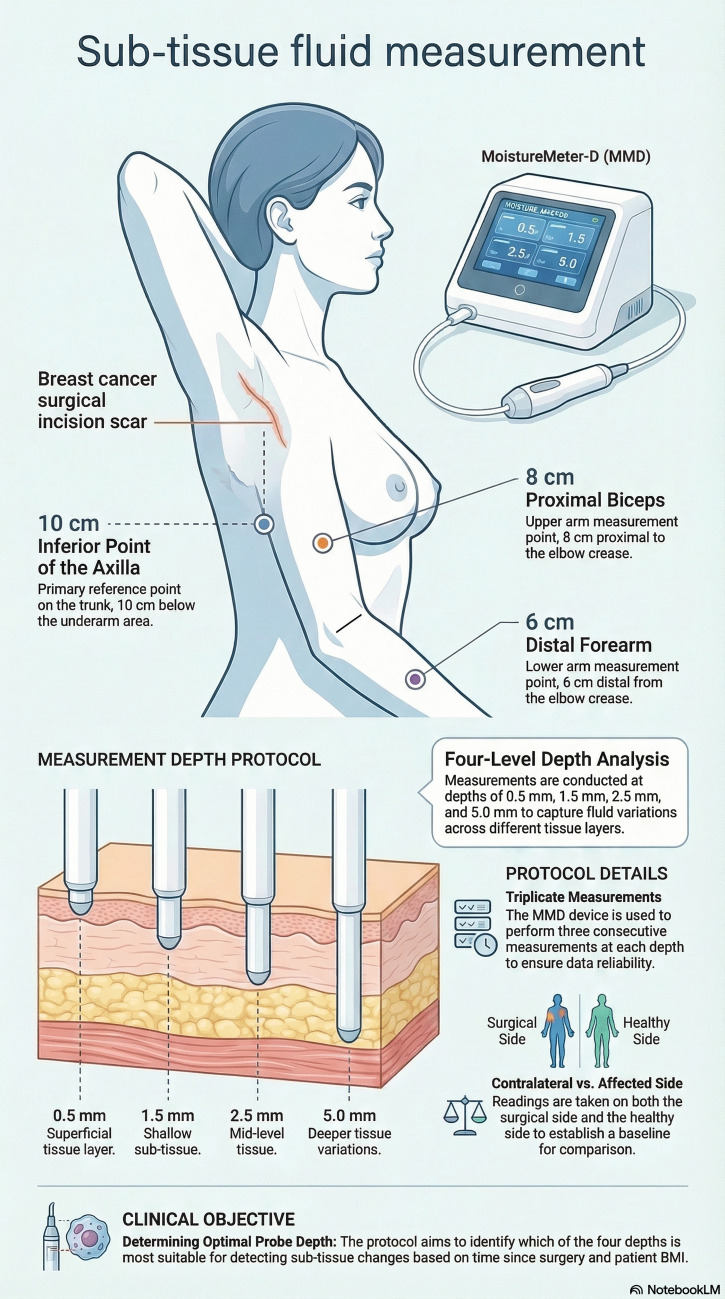
Schematic illustration of tissue dielectric constant (TDC) measurement. The infographic was created by the authors using NotebookLM (Google) and subsequently edited for clarity

The tissue dielectric constant (TDC) was measured via MoistureMeterD (Delfin Technologies, Kuopio, Finland) in the following four predefined measurement points in both upper extremities: 10 cm proximal and 6 cm distal medial points for the upper arm and forearm, the dorsum of the web space, and an 8-cm distal point from the axillary space. The working procedure of the MoistureMeterD was well explained previously [23–25]. Briefly, the probe that is in contact with the skin transmits the 300-MHz electromagnetic wave generated in the control unit. The reflected signal is processed in the control unit and gives the dielectric value of the measurement site. Pure water has a dielectric value of 78, while air has 1 at room temperature. The higher the dielectric value represents the higher the fluid content, or vice versa. The reflected part is mostly based on the fluid content of the tissue, and it is attributed to the edema and/or extracellular fluid. MoistureMeterD has four different probes which have unique effective penetration depths and contact diameters as follows: 0.5 (10 mm), 1.5 (20 mm), 2.5 (23 mm), and 5.0 (55 mm). Visual representation of the measurement procedure of TDC is shown in Fig. 1.

Patients who were lying supine for at least 5 min after cleaning and marking the measurement sites with a soft pen were measured with each probe in all reference points (arm, forearm, thorax, and web space). Precautions were taken prior to the measurement as there needed to be no cream or lotion(s) on the measurement site. Three measurements were taken for each point and their mean was recorded as TDC. All procedures were tried to be performed at a 25 °C room temperature and constant humidity. TDC assessment was performed prior to CT and within a week of the end of the last CT session.

### Statistical analysis

The data was shown as means and standard deviation or numbers and percentages according to the type of data. Normality of continuous variables was assessed using the Shapiro–Wilk test and the Kolmogorov–Smirnov test, along with evaluation of skewness and kurtosis values. Since the design of this study was pre- and post-test, a paired sample *t*-test was used to analyze TDC values for each reference point. In case of a violated assumption of normality, the Wilcoxon signed-rank test was used. Dichotomous variables were analyzed via the chi-square test. All analyses were two-tailed and the *p*-value was accepted as significant below 0.05. Statistical analysis was performed with IBM SPSS v.20 (IBM Corp, NY).

According to the post hoc power analysis performed via GPower 3.1 [26], when considering the TDC ratio of web space reference point collected via a 2.5-mm depth probe according to the paired *t*-test in a total of 51 patients, we achieved 0.93 power with a medium effect size (Cohen’s *d* = 0.5).

## Results

A total of 56 patients with BC who will undergo systemic taxane-based chemotherapy were screened and invited to participate in this study. However, five patients could not be measured for some reasons both in pre-CT and post-CT. Therefore, this study was completed with a total of 51 patients (mean age and BMI, 51.45 ± 11.67 years and 28.41 ± 5.1 kg/m^2^). Twelve and 39 patients were referred to neoadjuvant chemotherapy (NACT) and adjuvant chemotherapy (ACT), respectively. Anthracycline and paclitaxel were applied in 28 out of 51 patients, while the rest of them directly underwent docetaxel. The clinical and sociodemographic characteristics of patients are shown in Table 1.

**Table 1:** Sociodemographic and clinical characteristics of patients

	*n* = 51	
	*n* = 39	*n* = 12
	ACT	NACT
Marital status		
Married	28	8
Single or divorced	11	4
Occupation		
Active working	15	9
Not working	24	3
Chemotherapy protocol		
AC + PAXL	18	11
TC (Docetaxel)	21	1
Type of breast surgery		
Conservative	30	-
MRM	9	-
Axillary procedure		
ALND	32	-
SLNB	7	-
Stage^*^		
T1	9	-
T2	22	4
T3	8	8

*ACT* adjuvant chemotherapy, *NACT* neo-adjuvant chemotherapy, *AC* adriamycin + cyclophosphamide, *PAXL* paclitaxel, *TC* taxotere + cyclophosphamide (docetaxel), *MRM* modified radical mastectomy, *ALND* axillary lymph node dissection, *SLNB* sentinel lymph node biopsy

^*^Staging was performed via histopathological staging for ACT patients or clinical staging for NACT patients

In the total group, the web space reference point in the affected site was found to have significantly higher TDC values in 0.5-, 1.5-, and 2.5-mm depths after CT. There was no significant change concerning TDC values as well as TDC ratios after CT except for the forearm reference point which had a significantly higher TDC value after CT in the 1.5 mm probe (t = −2.17, *p* = 0.03) (Table 2). In the ACT group, the web space reference point was found to have significantly higher TDC value in 0.5-, 1.5-, and 2.5-mm probe depths after CT. The forearm reference point had a significantly higher TDC value in 1.5- and 2.5-mm probes after CT, while the arm reference point had a significantly higher TDC value only in 1.5 mm (Table 3). In the NACT group, no significant change(s) was found before and after CT in all reference points except for the affected site (tumor site) thorax reference point which had a significantly higher TDC value and TDC ratio in the 5.0-mm probe after CT. In addition, a significant increase in the TDC ratio was also observed in the 1.5-mm probe in the NACT group (Table 4). Although changes in TDC values differed significantly, no significant difference was observed in TDC ratios in all reference points among all probes.

**Table 2:** TDC values and ratios of affected and unaffected sites of patients before and after chemotherapy

*n* = 51 TDC	Pre-CT			Post-CT			Within group comparisons (affected sites only)	
	Aff	Unf	Ratio	Aff	Unf	Ratio	*t*	*p*
0.5 mm								
Arm	35.05 ± 4.33	33.95 ± 3.45	1.03 ± 0.09	35.99 ± 5.88	34.24 ± 4.14	1.05 ± 0.12	− 1.56	.125
Forearm	36.35 ± 5.05	36.04 ± 4.13	1.00 ± 0.08	37.35 ± 5.91	36.76 ± 4.26	1.01 ± 0.10	− 1.54	0.13
Web space	36.04 ± 3.97^*^	35.80 ± 3.52	1.00 ± 0.06	37.44 ± 4.27^*^	36.60 ± 3.79	1.02 ± 0.09	− 2.21	**.032**
Thorax	37.20 ± 5.99	35.78 ± 4.09	1.04 ± 0.13	37.02 ± 5.16	35.94 ± 4.15	1.03 ± 0.09	0.31	.75
1.5 mm								
Arm	30.35 ± 4.50	30.03 ± 3.69	1.01 ± 0.11	31.70 ± 5.94	30.56 ± 3.71	1.04 ± 0.16	− 1.88	.06
Forearm	31.60 ± 4.06^*^	31.60 ± 3.74	1.00 ± 0.08	32.98 ± 5.29^*^	32.42 ± 4.08	1.02 ± 0.10	− 2.16	**0.03**
Web space	33.70 ± 4.03^*^	33.52 ± 3.51	1.00 ± 0.10	34.98 ± 3.81^*^	34.55 ± 3.83	1.02 ± 0.10	− 2.17	**0.03**
Thorax	33.11 ± 5.05	31.74 ± 3.79	1.05 ± 0.15	32.91 ± 5.21	31.77 ± 3.87	1.04 ± 0.13	.592	.55
2.5 mm								
Arm	26.94 ± 3.43	25.97 ± 3.03	1.04 ± 0.09	27.27 ± 4.28	26.34 ± 3.11	1.03 ± 0.11	−.719	.47
Forearm	27.90 ± 3.68	27.75 ± 2.85	1.00 ± 0.07	28.79 ± 4.52	28.41 ± 3.21	1.01 ± 0.10	− 1.65	.10
Web space	32.79 ± 3.90^*^	32.88 ± 3.45	0.99 ± 0.08	34.68 ± 4.22^*^	33.90 ± 3.50	1.02 ± 0.10	− 3.59	**.001**
Thorax	29.66 ± 4.19	28.37 ± 3.20	1.05 ± 0.10	29.74 ± 3.86	28.71 ± 3.23	1.04 ± 0.10	−.024	.98
5.0 mm								
Arm	20.38 ± 4.40	19.77 ± 2.42	1.03 ± 0.20	21.04 ± 3.68	20.28 ± 2.75	1.04 ± 0.15	− 1.01	.31
Forearm	21.67 ± 3.87	21.21 ± 2.77	1.02 ± 0.11	22.42 ± 4.02	21.73 ± 2.85	1.03 ± 0.16	− 1.07	.28
Web space	-	-	-	-	-	-	-	-
Thorax	22.56 ± 3.47	21.56 ± 2.75	1.05 ± 0.14	23.60 ± 3.64	21.96 ± 2.80	1.08 ± 0.12	− 1.72	.92

*Aff* affected, *Unf* unaffected, *mm* millimeter, *CT* chemotherapy

^*^The affected site was chosen for the tumor site for patients receiving neoadjuvant chemotherapy

**Table 3:** TDC values and ratios of affected and unaffected sites of patients who underwent adjuvant chemotherapy

*n* = 39 TDC	Pre-CT				Post-CT			
	Aff	Unf	Ratio	Ratio (± 2SD)	Aff	Unf	Ratio	Ratio (± 2SD)
0.5 mm								
Arm	35.36 ± 4.48	33.98 ± 3.66	1.04 ±.10	1.24	37.08 ± 5.89	34.57 ± 4.28	1.07 ±.13	1.33
Forearm	36.67 ± 5.39	36.30 ± 4.37	1.01 ±.08	1.17	38.25 ± 6.06	37.39 ± 4.17	1.02 ±.11	1.24
Web space	35.85 ± 3.98^*^	35.40 ± 3.47	1.01 ±.06	1.13	38.24 ± 4.00^*^	36.85 ± 3.66	1.04 ±.10	1.24
Thorax	37.40 ± 6.53	35.46 ± 4.39	1.05 ±.14	1.33	36.99 ± 5.40	35.74 ± 4.29	1.03 ±.10	1.23
1.5 mm								
Arm	30.67 ± 4.57^*^	29.98 ± 3.87	1.02 ±.11	1.24	32.68 ± 5.79^*^	30.67 ± 3.91	1.07 ±.15	1.37
Forearm	31.80 ± 4.39^*^	31.73 ± 3.91^*^	1.00 ±.08	1.16	33.76 ± 5.46^*^	33.04 ± 4.02^*^	1.02 ±.10	1.22
Web space	33.44 ± 3.91^*^	33.10 ± 3.52	1.01 ±.11	1.23	35.49 ± 3.88^*^	34.62 ± 3.64	1.03 ±.11	1.25
Thorax	33.19 ± 5.49	31.48 ± 4.06	1.06 ±.16	1.38	32.71 ± 5.54	31.57 ± 3.79	1.04 ±.15	1.34
2.5 mm								
Arm	27.27 ± 3.56	26.09 ± 3.26	1.05 ±.10	1.25	27.99 ± 4.37	26.49 ± 3.27	1.06 ±.12	1.30
Forearm	28.29 ± 3.85^*^	28.11 ± 2.93	1.00 ±.08	1.16	29.56 ± 4.62^*^	28.79 ± 3.23	1.03 ±.10	1.23
Web space	32.56 ± 3.82^*^	32.69 ± 3.49^*^	0.99 ±.09	1.17	35.14 ± 4.27^*^	34.20 ± 3.21^*^	1.03 ±.11	1.25
Thorax	29.93 ± 4.47	28.20 ± 3.40	1.06 ±.11	1.28	29.74 ± 4.21	28.50 ± 3.29	1.04 ±.10	1.24
5.0 mm								
Arm	21.16 ± 3.33	19.83 ± 2.58	1.07 ±.14	1.35	21.63 ± 3.69	20.32 ± 2.84	1.07 ±.15	1.37
Forearm	22.02 ± 4.09	21.46 ± 2.82	1.02 ±.11	1.24	22.83 ± 4.13	21.89 ± 3.01	1.05 ±.17	1.39
^⸷^Web space	-	-	-		-	-	-	
Thorax	22.88 ± 3.54	21.40 ± 2.89	1.07 ±.13	1.33	23.46 ± 3.90	21.76 ± 2.66	1.08 ±.12	1.32

*Aff* affected, *Unf* unaffected, *mm* millimeter

^*^Significant at 0.05 level before and after CT within the same sides

^⸷^Webspace could not be measured with a 5.0-mm probe due to the unconformity between the probe and the area

**Table 4:** TDC values and ratios of affected and unaffected sites of patients who underwent neoadjuvant chemotherapy

*n* = 12 TDC	Pre-CT				Post-CT			
	Aff	Unf	Ratio	Ratio (± 2SD)	Aff	Unf	Ratio	Ratio (± 2SD)
0.5 mm								
Arm	33.57 ± 3.38	33.81 ± 2.37	0.99 ±.06	1.11	31.87 ± 3.74	33.00 ± 3.44	0.97 ±.04	1.05
Forearm	34.86 ± 2.71	34.81 ± 2.53	1.00 ±.04	1.08	33.94 ± 3.91	34.34 ± 3.84	0.99 ±.03	1.05
Web space	36.99 ± 4.06	37.70 ± 3.32	0.98 ±.04	1.06	34.39 ± 4.07	35.66 ± 4.32	0.96 ±.05	1.06
Thorax	36.26 ± 2.04	37.27 ± 1.71	0.97 ±.04	1.05	37.15 ± 4.39	36.71 ± 3.64	1.01 ±.09	1.19
1.5 mm								
Arm	28.85 ± 4.07	30.30 ± 2.91	0.95 ±.08	1.11	27.99 ± 5.23	30.15 ± 2.96	0.93 ±.15	1.23
Forearm	30.70 ± 1.83	31.01 ± 2.91	0.99 ±.11	1.25	30.05 ± 3.36	30.08 ± 3.61	1.00 ±.10	1.20
Web space	34.96 ± 4.61	35.51 ± 2.88	0.98 ±.06	1.10	33.07 ± 2.96	34.33 ± 4.71	0.97 ±.08	1.13
Thorax	32.73 ± 2.08	33.00 ± 1.75	0.99 ±.04^*^	1.07	33.66 ± 3.85	32.56 ± 4.27	1.04 ±.09^*^	**1.22**
2.5 mm								
Arm	25.40 ± 3.37	25.37 ± 1.66	1.00 ±.06	1.12	24.52 ± 2.62	25.78 ± 2.52	0.95 ±.05	1.05
Forearm	26.06 ± 2.03	26.07 ± 1.77	1.00 ±.05^*^	1.10	25.87 ± 2.70	27.01 ± 2.84	0.96 ±.06^*^	**1.08**
Web space	33.86 ± 4.38	33.76 ± 3.36	1.00 ±.06	1.12	32.94 ± 3.72	32.77 ± 4.44	1.00 ±.06	1.12
Thorax	28.41 ± 2.21	29.15 ± 1.92	0.97 ±.06	1.09	29.76 ± 2.23	29.51 ± 3.04	1.01 ±.08	1.17
5.0 mm								
Arm	17.07 ± 6.70	19.51 ± 1.63	0.88 ±.34	1.56	18.79 ± 2.76	20.12 ± 2.49	0.93 ±.07	1.07
Forearm	20.02 ± 2.16	20.07 ± 2.33	1.00 ±.09	1.18	20.89 + 3.29	21.14 ± 2.17	0.98 ±.06	1.10
Web space	-	-	-	-	-	-	-	-
Thorax	21.10 ± 2.88^*^	22.35 ± 1.94	0.94 ±.11^*^	**1.16**	24.13 ± 2.48^*^	22.72 ± 3.36	1.07 ±.13^*^	**1.33**

*Aff* affected, *Unf* unaffected, *mm* millimeter, *CT* chemotherapy

^*^The affected site was chosen for the tumor site for patients receiving neoadjuvant chemotherapy

Changes in TDC values and ratios between docetaxel and combined anthracycline and paclitaxel groups were analyzed only in the ACT group. There was no significant difference in TDC values and ratios gathered via four different probes in all reference points after CT. Although we had a small number of patients who underwent sentinel lymph node biopsy, there was no significant difference between patients with ALND or SLNB in terms of TDC values and ratios in all reference points before and after CT.

## Discussion

The present study showed that although there were significant increases in TDC values taken in varied probes in some of the reference points in the affected sites after CT in the adjuvant taxane-based CT group, no significant difference was observed in the TDC ratio(s). Although some TDC ratios increased by nearly the rate of 10% after CT, these changes did not reach statistical significance. Those are consistent with the previous findings, in which the indicator of having BCRL according to the threshold of the TDC ratio is above 1.4 [15]. Thus, using the TDC ratio might be more objective to be used for detecting potential BCRL, especially in patients undergoing systemic therapy. Yet, it might be reasonable to say that the acute effects of taxanes cannot be elucidative of potential BCRL in the perspective of sub-tissue fluid proportions according to our study findings.

Taxanes have been known to be effective in the treatment of BC by increasing survival rates. Although this systemic treatment has become a standard of care for BC treatment, it is also well known for its varied types of side effects [27, 28]. Though the contribution of taxanes in terms of BCRL is still under debate, this class of drugs is responsible for edema by acting to increase capillary permeability; therefore, fluid accumulation occurs [29]. It was reported that BC patients who were treated with taxanes experienced peripheral edema [9]. Ohsumi et al. [30] also reported that BC patients treated with taxanes suffer from peripheral edema which lasted over 12 months from the termination of taxane therapy. Albeit there are many factors related to the manifested BCRL, Cariati et al. [31] reported that adjuvant taxane-based chemotherapy was the strongest factor in the development of BCRL. Lee et al. [27] also reported elevated extracellular fluid according to the bioimpedance spectroscopy, which corresponds to the clinical BCRL up to 6 months after taxane-based chemotherapy in the affected site. However, we did not find a clinically relevant ratio according to the dielectric values. Yet, significantly increased TDC values, especially in the affected site’s web space reference point, may support the potential role of taxanes on peripheral edema specifically in 1.5- and 2.5-mm measurement depths in our adjuvant CT group. Our increased TDC values after CT in the ACT group were also in parallel with the findings that increased load on lymphatics due to taxanes, especially in the case of axillary node dissection [32]. Since the majority of our sample underwent ALND (32 out of 39), this increase should be interpreted not only with the pure effect of Taxanes but also with the type of axillary dissection. Although we had a small sample of the NACT group, there was no significant difference in terms of TDC values after CT, which should be considered that taxanes might not be the only factor in terms of the development of BCRL. Yet, the significantly increased TDC value and ratio of the affected site’s thorax reference point in the deepest probe (5.0 mm) might be explained by the possible accumulation of drugs close to the peritumoral site in the NACT group. On the other hand, Jung et al. [11] reported that taxanes, as well as NACT, were found to be independent risk factors for BCRL. However, they reported the retrospective data of patients who already had curative surgery along with axillary dissection. We did not measure the TDC values of our patients who underwent NACT after surgery; therefore, we cannot conclude on the potential effect of taxanes on BCRL among patients who underwent NACT. Yet, Swaroop et al. [9] reported that taxanes did not act a role in the development of BCRL. Nevertheless, they used the relative volume change of > 10% as a diagnostic criterion of BCRL. Considering the prolonged effect of generalized edema due to systemic chemotherapy, we think that this criterion might not be sensitive enough to detect BCRL. Despite no significant difference being achieved after CT in patients who underwent NACT in our study, those should be strictly monitored after surgery since Johnson et al. [32] reported that mean lymphatic contractility was lower in patients who had NACT therapy. When it comes to the peripheral or unaffected site edema, Lee et al. [27] reported an increase of edema in the lower extremities and unaffected upper extremity, which was resolved after 6 months of the completion of the chemotherapy. In our study, we did not measure the lower extremities. Yet, unaffected sites TDC values were not changed except for forearm and web space reference points only in 1.5- and 2.5-mm measurement depths after CT. As we mentioned earlier, these changes did not affect the inter-arm ratio, which should be taken into account when considering potential BCRL.

There are various methods to detect BCRL. Tape measurement, volumetric change, BIS, and TDC have been reported to detect BCRL along with symptomatic assessment. However, since some parameters may fail to be detected sensitively or are over or underestimated, the incidence of BCRL may remarkably vary [12]. Since the TDC method can be quite useful to be used locally wherever needed, this method was reported to be a promising modality [14, 16, 20, 33]. Yet, when interpreting the changes of TDC associated with BCRL, some points should be undertaken. For instance, Mazor et al. [33] reported that TDC values were significantly higher in the affected site compared to the unaffected site in all reference points except for web space in patients with lymphedema. In this case, it should be noted that the localization of lymphedema might have played a major role. Mayrovitz et al. [34] reported earlier that the threshold of the inter-arm TDC ratio of 1.2 can be useful for detecting potential BCRL. We also found that the specificity of the TDC method was over 90% in our previous study according to the 1.2 threshold when comparing the inter-arm ratio of patients with and without BCRL [7]. Though mean actual ratios were found under 1.2 in this study, in some reference points, this threshold was exceeded when adding two standard deviations. Since some reference points such as the forearm were reported to be the most useful sites to detect early BCRL, we think that patients who present an actual inter-arm TDC ratio over 1.2 should be closely monitored along with symptomatologic assessment. Our ACT group affected site forearm TDC values were significantly increased after CT in 1.5- and 2.5-mm measurement depths; however, ratios did not change significantly (1.16 to 1.22 [< 10%], 1.17 to 1.25 [< 10%]; see Table 3). Yet, these thresholds were reported for the upper extremities. For instance, inter-breast TDC ratios of 1.28 [35] or 1.4 [36] were reported to be useful in detecting breast edema and/or lymphedema.

The present study contributes to the growing body of literature investigating objective methods for detecting early tissue fluid changes in patients undergoing breast cancer treatment. Although early detection of taxane-induced edema using TDC measurements was not observed in our sample, the findings provide important preliminary insights into the pattern of sub-tissue fluid changes during taxane-based chemotherapy. To our knowledge, prospective data evaluating TDC measurements specifically in patients receiving taxane-based chemotherapy remain limited. Therefore, our results may help clarify the potential role and limitations of TDC assessment in monitoring chemotherapy-related edema and may guide future studies investigating early detection strategies for BCRL. Importantly, the absence of detectable early fluid changes in the present study may also highlight the complexity of chemotherapy-related fluid alterations and suggest that TDC measurements alone may not be sufficiently sensitive to identify early subclinical edema in this population. These findings underscore the need for future studies incorporating larger cohorts, longitudinal follow-up, and multimodal assessment approaches combining objective measurement tools with clinical and patient-reported outcomes. Such research may help clarify the optimal strategies for early detection and monitoring of chemotherapy-related edema and BCRL.

This study has some strengths and limitations. We have included patients from a single outpatient clinic and all our patients were white Caucasian women. Therefore, the generalizability of our results might be a bit arguable. Second, we did not assess patients’ symptoms regarding BCRL as well, and we did not assess circumferential or volumetric measures of extremities. Thus, a comparison of potential preclinical BCRL is lacking. We also did not measure our patients in a follow-up period. Group-based imbalanced sample sizes may have also influenced the statistical results; therefore, this could be added as a limitation of the study. Yet, the prospective nature and measuring and tracking patients on a strict timeline can be accepted as strengths of this study. It should also be noted that the TDC method has been studied by limited researchers; therefore, there is a need to expand and compare the knowledge regarding the detection of potential BCRL.

## Conclusion

Breast cancer-related lymphedema (BCRL) is the most debilitating and chronic condition after breast cancer treatment [3]. Due to the chronic nature of this situation, early detection of the BCRL can improve clinical outcomes. Since breast cancer treatment shows itself with a multimodal treatment, tracking and monitoring the changes associated with the BCRL need more sensitive assessments. In this study, it was shown that taxane-based chemotherapy did not affect the potential BCRL in the completion of systemic treatment in acute settings. However, longer follow-ups are needed to confirm these results, especially in patients who underwent taxane-based chemotherapy along with carrying other reported risk factors according to the literature.

## Data Availability

The data can be available from the corresponding author upon reasonable request and with permission of Bakircay University Ethical Board of Clinical Studies.
